# Association Between Peak Body Mass Index and Experienced Weight Stigma Among Adults With Obesity

**DOI:** 10.1002/osp4.70202

**Published:** 2026-11-09

**Authors:** Aparajita Chandrasekhar, Deepali K. Ernest, Jane Guo, Gloria L. Vega, Matthew Cali, Luyu Xie, Sophia Ngenge, Jeffrey N. Schellinger, Matthew Sunil Mathew, Sarah E. Messiah, Jaime P. Almandoz

**Affiliations:** ^1^ Department of Epidemiology, Human Genetics, and Environmental Sciences University of Texas Health Science Center at Houston School of Public Health Houston Texas USA; ^2^ Department of Clinical Nutrition UT Southwestern Medical Center School of Health Professions Dallas Texas USA; ^3^ UT Southwestern Medical Center Medical School Dallas Texas USA; ^4^ Department of Public Health UT Southwestern Medical Center Peter O'Donnell Jr. School of Public Health Dallas Texas USA; ^5^ Department of Pediatrics UT Southwestern Medical Center Dallas Texas USA; ^6^ Children's Health System of Texas Dallas Texas USA; ^7^ Department of Internal Medicine Division of Endocrinology UT Southwestern Medical Center Dallas Texas USA

**Keywords:** body mass index, obesity, weight stigma

## Abstract

**Background:**

Experienced weight stigma (WS) is associated with negative health and psychosocial outcomes; however, the association of lifetime peak body mass index (BMI) with experienced WS remains unclear. This study examines the association between peak BMI and experienced WS.

**Methods:**

Cross‐sectional survey data from 794 patients in an academic obesity medicine program were analyzed. Experienced WS was assessed using the Stigmatizing Situations Inventory—Brief and was examined as a continuous variable. Multivariable linear regression analysis performed the association between peak BMI and experienced WS, adjusting for demographics and bariatric surgery (MBS) history.

**Results:**

Participants (mean age: 56.2 years, 86.3% female) had a mean peak BMI of 44.3 kg/m^2^. More frequent experienced WS was more prevalent in those with peak BMI ≥ 40 kg/m^2^ versus < 40 kg/m^2^ (58.5% vs. 34.1%, *p* < 0.001). After adjustment, per‐unit increase in peak BMI was associated with a 0.54 experienced WS score increase (β = 0.54; 95% CI: 0.43, 0.65; *p* < 0.001). MBS history was associated with increased experienced WS score, while older age and Non‐Hispanic Black were associated with lower WS score.

**Conclusion:**

Increasing peak BMI is associated with higher experienced WS scores, independent of current BMI. Clinicians should consider weight history to identify at‐risk individuals and inform stigma‐reducing interventions.

## 1 Introduction

Weight stigma is defined as antagonistic attitudes, behaviors, and stereotypes directed toward individuals due to their body weight, shape, or size [1]. Experienced weight stigma (WS) refers to the negative experiences characterized by antagonistic gestures which can contribute to marginalization or prejudice among those with obesity [2, 3]. A significant proportion (42%) of United States (US) adults reported experienced WS, regardless of weight status [4]; and research has found that experienced WS among individuals with obesity is associated with negative health outcomes, such as a higher risk for cardiovascular disease, greater calorie intake, and increased body mass index (BMI) [5, 6]. Experienced WS is linked to adverse psychological outcomes, including increased risks of anxiety, depression, suicidality, poorer health‐related quality of life, body dissatisfaction, and heightened emphasis on physical appearance [6].

Although there is a known association between experienced WS and living with obesity, little is known about the association between experienced WS and a person's peak lifetime BMI. Specifically, a peak BMI ≥ 40 kg/m^2^ (class III obesity), which has increased in prevalence to impact almost 1 in 10 adults in the United States [7, 8]. Additionally, a 2016 meta‐analysis found that individuals with more severe obesity (i.e., BMI ≥ 35 kg/m^2^) had a prevalence of weight‐based discrimination of approximately 42%, as compared to 19% among individuals with class I obesity (BMI 30–34.9 kg/m^2^) [9]. As such, the increased prevalence of weight‐based discrimination among those with higher BMI underscores the importance of determining the association between peak BMI and experienced WS. Experienced WS is associated with a multitude of maladaptive psychological health implications. Previously conducted research strongly suggests that with respect to mental health outcomes, it is the obesity stigmatization (i.e., experienced WS) that exacerbates the risk of developing depression. A meta‐analysis comprised of 105 studies found that weight‐based stigma was significantly associated with poorer mental health outcomes (*r* = −0.35; *p* < 0.001) [10].

Aside from the mental health implications of experienced WS, it is also associated with short‐ and long‐term physical health implications. Building upon the established association between higher BMI and increased experienced WS, it is important to consider that experienced WS itself may increase one's risk of having obesity through a few plausible mechanisms [10]. Possible mechanisms include experienced WS making it difficult for individuals with obesity to lose weight, avoidance of certain physical activity settings, and medication non‐adherence [10]. As such, elucidating the relationship between peak BMI and experienced WS is critical given that peak BMI may already be representative of more frequent exposure to WS and since obesity stigmatization may further compound one's obesity and related comorbidities.

While a patient may not be at their peak BMI at the time of a clinical encounter, it may be important to take a comprehensive weight history to assess their risk of EWS and other obesity‐related complications. These data could help clinicians and health systems to identify patients who may benefit from interventions to address experienced WS and optimize health outcomes. As such, this study aimed to investigate whether there is an association between lifetime peak BMI and experienced WS.

## 2 Materials and Methods

### 2.1 Study Design

This study analyzed self‐reported cross‐sectional survey data that included demographics, medical history, and social/self‐perceptions collected from patients of an academic center's obesity medicine program between May 2022 and August 2022.

### 2.2 Subjects

A total of 1075 participants enrolled in the study, with complete survey data available for 794 individuals included in the final analysis.

### 2.3 IRB Approval

The University of Texas Southwestern Medical Center Institutional Review Board approved the study (IRB# STU‐2022‐0265).

### 2.4 Main Outcome Variable

The main outcome variable was experienced WS, modeled as a continuous variable in regression analyses. This study used the Stigmatizing Situations Inventory—Brief (SSI‐B) to assess EWS. The SSI‐B questionnaire is a standardized and reliable measure used to quantify weight stigma among individuals with obesity and a history of metabolic bariatric surgery (MBS) [11, 12]. The SSI‐B is comprised of 10 items and uses a 10‐point Likert scale for each item (0‐9; e.g., 0 = never, 1 = once in your life, 2 = several times in your life, 3 = about once a year, 4 = several times per year, 5 = about once a month, 6 = several times per month, 7 = about once a week, 8 = several times per week, and 9 = daily) with a possible total score range of 0–90.

### 2.5 Main Exposure

The main exposure variable was lifetime peak BMI and participants were stratified by BMI status for class III obesity (BMI ≥ 40 or < 40 kg/m^2^). Lifetime peak BMI was modeled as a continuous variable in regression analyses.

### 2.6 Covariates

Covariates included age, sex, race/ethnicity, MBS status, and education. In the adjusted linear regression model, all covariates were modeled as categorical variables except for age, which was modeled as a continuous variable. Sex was categorized as female or male (reference group). Race/ethnicity was categorized into 4 groups: non‐Hispanic Black, Hispanic, Other, and non‐Hispanic White (reference group). Bariatric surgery status was categorized as MBS completer versus non‐MBS (reference group). Education was categorized into 4 groups: high school or less (reference group), trade school, bachelor's, and master's or higher.

### 2.7 Statistical Analysis

Descriptive analysis was conducted for baseline demographics such as age, sex, race/ethnicity, current BMI, peak BMI, experienced WS score, and MBS completion status. Multivariable linear regression assessed the association of peak BMI and experienced WS to demonstrate the change in experienced WS stigma score per‐unit increase in peak BMI, adjusting for covariates such as age, sex, race/ethnicity, MBS status, and education. All statistical analyses were performed using STATA/BE 15.1 (StataCorp, College Station, Texas). Significance was determined via a two‐sided *p*‐value < 0.05.

## 3 Results

The final analytical sample consisted of 794 participants, with a mean age of 56.21 years (SD: 12.59), lifetime peak BMI 44.34 kg/m^2^ (SD: 11.33), and current BMI 34.54 kg/m^2^ (SD: 9.65). In this sample, 60.45% reported a lifetime peak BMI ≥ 40 kg/m^2^ and 22.29% had a current BMI ≥ 40 kg/m^2^. Most of the sample was female (86.28%) with diverse representation from non‐Hispanic Black (NHB) (20.65%) and Hispanic groups (13.85%). The median SSI‐B score for the sample was 11.00.

When stratified by lifetime peak BMI category (< 40 vs. ≥ 40 kg/m^2^), significant demographic differences were observed (Table 1). Participants with a peak BMI ≥ 40 kg/m^2^ had a higher mean peak BMI (50.54 (SD: 10.37) versus 34.86 kg/m^2^ (SD: 3.35)) and higher current BMI (38.21 (SD: 10.33) versus 28.92 kg/m^2^ (SD: 4.49)) compared to those with a lifetime peak BMI < 40 kg/m^2^. Additionally, individuals in the peak BMI ≥ 40 kg/m^2^ group had a greater proportion of non‐Hispanic Black (24.79% vs. 14.33%) and Hispanic participants (15.00% vs. 12.10%), whereas non‐Hispanic White participants were more prevalent in the peak BMI < 40 kg/m^2^ group (67.83% vs. 55.63%). Regarding weight stigma, the median SSI‐B score was significantly higher among those with a lifetime peak BMI ≥ 40 kg/m^2^ (17.50 vs. 5.00). Differences were also observed in metabolic and bariatric surgery (MBS) status, with 54.58% of participants in the peak BMI ≥ 40 kg/m^2^ group having undergone MBS, compared to only 8.60% in the peak BMI < 40 kg/m^2^ group. These findings suggest that individuals with a lifetime peak BMI ≥ 40 kg/m^2^ have higher current BMIs and have undergone MBS, emphasizing the long‐term impact of lifetime peak BMI on obesity‐related stigmatizing experiences and MBS utilization.

**TABLE 1:** Demographic characteristics of the study sample (*N* = 794) stratified by lifetime peak BMI status.

Demographics	Overall (*N* = 794)	Peak BMI < 40 (*N* = 314)	Peak BMI ≥ 40 (*N* = 480)	*p*‐value
Age (years) mean (SD)	56.21 (12.59)	57.86 (13.18)	55.13 (12.01)	0.003
Sex *N* (%)				
Male	109 (13.72)	45 (14.33)	64 (13.33)	0.690
Female	685 (86.28)	269 (85.67)	416 (86.67)	
Race and ethnicity *N* (%)				
Non‐Hispanic white	480 (60.45)	213 (67.83)	267 (55.63)	0.001
Non‐Hispanic black	164 (20.65)	45 (14.33)	119 (24.79)	
Hispanic	110 (13.85)	38 (12.10)	72 (15.00)	
Other	40 (5.04)	18 (5.73)	22 (4.58)	
BMI^a^ (kg/m^2^) mean (SD)				
Current BMI	34.54 (9.65)	28.92 (4.49)	38.21 (10.33)	< 0.001
Peak lifetime BMI	44.34 (11.33)	34.86 (3.35)	50.54 (10.37)	< 0.001
EWS^b^ (median SSI‐B score)	(11.00)	(5.00)	(17.50)	< 0.001
MBS^c^ status *N* (%)				
Non‐MBS	505 (63.60)	287 (91.40)	218 (45.42)	< 0.001
MBS completer	289 (36.40)	27 (8.60)	262 (54.58)	
Education *N* (%)				
High school of less	168 (21.29)	66 (21.22)	102 (21.34)	0.104
Trade school	80 (10.14)	22 (7.07)	58 (12.13)	
Bachelor's degree	283 (35.87)	112 (36.01)	171 (35.77)	
Master's degree or higher	258 (32.70)	111 (35.69)	147 (30.75)	

^a^ BMI: Body Mass Index.

^b^ Experienced WS: Experienced Weight Stigma.

^c^ MBS: Metabolic Bariatric Surgery.

Multivariable linear regression analysis demonstrated that per‐unit increase in lifetime peak BMI was significantly associated with a 0.54 experienced WS score increase (β = 0.54; 95% CI: 0.43, 0.65; *p* < 0.001) (Table 2). Each 1‐year increase in age was significantly associated a 0.25 score decrease (β = ‐0.25; 95% CI: −0.33, −0.15; *p* < 0.001). Race/ethnicity was a significant predictor: compared to non‐Hispanic White participants, non‐Hispanic Black participants showed a 5.70 score decrease (β = ‐5.70; 95% CI: −8.60, −2.81; *p* < 0.001).

**TABLE 2:** Association between lifetime peak BMI and experienced weight stigma scores in multivariable linear regression models.

Parameter		Adjusted coefficient (95% CI)^a^	*p*‐value
Lifetime peak BMI^b^		0.54 (0.43, 0.65)	< 0.001
Age (years)		−0.25 (−0.33, −0.15)	< 0.001
Sex	Male	1.0 (ref)	—
	Female	−3.09 (−6.35, 0.16)	0.06
Race/ethnicity	Non‐Hispanic white	1.0 (ref)	
	Non‐Hispanic black	−5.70 (−8.60, −2.81)	< 0.001
	Hispanic	−2.91 (−6.34, 0.52)	0.10
	Other	4.14 (−1.11, 9.38)	0.12
MBS^c^ status	Non‐MBS	1.0 (ref)	—
	MBS completer	6.78 (4.07, 9.29)	< 0.001
Education	High school of less	1.0 (ref)	—
	Trade school	3.96 (−0.27, 8.19)	0.07
	Bachelor's	1.77 (−1.29, 4.83)	0.26
	Master's or higher	2.29	0.15

^a^ (95% CI): (95% Confidence Interval).

^b^ BMI: Body Mass Index.

^c^ MBS: Metabolic Bariatric Surgery.

Sex and education level were not significantly associated with experienced WS score. However, MBS completers were significantly more likely to have a 6.78 WS score increase compared to non‐MBS participants (β = 6.78; 95% CI: 4.07, 9.29; *p* < 0.001).

## 4 Discussion

In this study, we found that the per‐unit increase in lifetime peak BMI was significantly associated with 0.54 increase in experienced WS score. Additionally, those who had undergone MBS were significantly more likely to report higher WS scores compared with non‐MBS participants. These findings underscore the importance of considering peak BMI when assessing a patient's history of weight stigma and suggest that targeted interventions are needed to reduce the burden of EWS in high‐risk groups.

To our knowledge, this is the first study in the literature to describe the association between peak BMI and experienced WS. Understanding this relationship is important for two reasons—first, given the established association between higher BMI and stigmatizing experiences, an individual may have a heightened vulnerability to facing weight stigma at their peak BMI. Second, the growing prevalence of class III obesity (BMI of ≥ 40) and the negative health implications of experienced WS place individuals at a greater risk for cardiovascular disease, maladaptive health behaviors, and high BMI [5, 6].

As such, these findings highlight the need for clinicians to take a comprehensive weight history to determine peak BMI and screen at risk groups for experienced WS. This is vital since patients may not necessarily have class III obesity at the time of their visit but may have had a peak BMI of ≥ 40 at some point prior. Thus, taking a comprehensive weight history and screening at risk groups for experienced WS aids in providing insight into the potential negative health implications of experienced WS. Clinicians taking these two steps will allow targeted interventions to mitigate the physical and mental health consequences of experienced WS in people with obesity.

Experts who previously evaluated the relationship between weight stigma and health have recommended that weight stigma should be managed by healthcare providers within obesity care programs [13]. While not explicitly stated, it is reasonable to assume that the management of weight stigma by healthcare providers would entail both screening and treatment via interventions. Notably, Puhl and colleagues found that people with obesity and experienced WS were significantly less likely to seek healthcare when needed [14]. It is especially important for individuals with obesity, particularly those with class III obesity, who may be at their peak BMI, to seek healthcare as needed. This is given that they are at increased risk of complications such as type 2 diabetes, hypertension, and cancer that require screening and interdisciplinary care [14, 15, 16]. Healthcare avoidance by this patient population can create missed opportunities for screening and management of obesity‐related complications, which includes physical and mental health concerns.

Our results add to the growing body of literature linking higher body weight to greater experienced WS. However, the finding showing decreased experienced WS scores among non‐Hispanic Black participants compared with non‐Hispanic White participants warrants further investigation. Prior research suggests that cultural differences in body image perceptions and social resilience may influence how weight stigma is experienced and internalized [17, 18]. Non‐Hispanic Black communities may have greater acceptance of diverse body sizes [19], leading to lower internalization of weight‐related stigma. Alternatively, differences in healthcare interactions or coping mechanisms may contribute to these disparities [20]. Future studies should explore qualitative perspectives on weight stigma across racial and ethnic groups to clarify these patterns.

The strong association between MBS completion and reporting higher experienced WS is an important finding that can likely be explained by the high‐BMI qualification requirements necessary to undergo bariatric surgery (≥ 40 kg/m^2^ or >35 kg/m^2^ with the presence of an obesity‐related complication) at the time these participants completed MBS [21]. While MBS leads to significant weight loss, research suggests that stigma may persist even after weight reduction. Many individuals with severe obesity experience long‐standing stigma before undergoing surgery [22], which may shape their perceptions of weight‐related discrimination even after weight loss. Additionally, post‐surgical weight bias has been reported [23], where individuals who undergo MBS report judgment or unrealistic expectations about weight maintenance from healthcare providers and the public. Further research is needed to examine the persistence of experienced WS post‐MBS and to develop interventions that support MBS patients in coping with stigma.

This study had several strengths. It is among the first to examine the association between lifetime peak BMI and experienced weight stigma (experienced WS) and provides novel insights into how historical weight status, rather than current BMI alone, influences the experience of stigma. Additionally, this study included a racially and ethnically diverse sample, allowing for an analysis of differences in experienced WS across racial/ethnic groups. Understanding these differences is critical, as prior research suggests that perceptions and internalization of weight stigma may vary across racial/ethnic backgrounds due to cultural differences in body image, social resilience, and healthcare experiences. Furthermore, the study leveraged a validated measure of weight stigma (SSI‐B) and a clinical sample of patients engaged in obesity care, enhancing the relevance of findings for healthcare settings.

However, several limitations must be considered. One key limitation is the cross‐sectional design, which prevents the establishment of causal relationships between peak BMI and experienced WS. Although our findings suggest that increased lifetime peak BMI is significantly associated with higher experienced WS scores, longitudinal research is needed to assess how weight stigma evolves over time. Future studies should investigate whether early‐life stigma predicts long‐term health outcomes and whether weight loss and its modality mitigates or exacerbates experienced WS. Understanding the trajectory of experienced WS across different life stages could help identify critical periods for intervention and inform stigma‐reduction strategies.

Additionally, the reliance on self‐reported data introduces the possibility of recall and social desirability bias, particularly when participants report peak BMI and past experiences with stigma. Future research should incorporate qualitative approaches or longitudinal designs to explore how individuals experience and cope with experienced WS over time.

Our study population consisted of patients seeking care at an academic obesity medicine program, which may limit generalizability to the broader population. Individuals actively engaged in medical weight management may have different experiences with weight stigma, healthcare interactions, and weight trajectories compared with those not seeking clinical care. An additional limitation requiring consideration is that individuals seeking weight management treatment often have higher levels of existing weight bias internalization (WBI). As a result, this may strengthen the relationship between WS and downstream health implications due to high WBI, potentially increasing both sensitivity and detection of stigmatizing experiences. Future studies should examine experienced WS in community‐based populations, as well as in individuals with diverse weight loss histories, including those who have not pursued medical treatment for obesity.

Despite these limitations, our study provided important evidence supporting the need to incorporate comprehensive weight histories into clinical care. By recognizing that lifetime peak BMI is a significant predictor of experienced WS, clinicians can take steps to identify at‐risk individuals and implement targeted stigma‐reduction interventions. Furthermore, the ability to report on racial/ethnic differences in experienced WS enhances our understanding of how stigma is experienced across diverse populations. Future research should further explore how sociocultural factors shape experienced WS and develop evidence‐based strategies to mitigate weight stigma, particularly in healthcare settings where it may contribute to healthcare avoidance and adverse patient outcomes.

## 5 Conclusion

This study demonstrated that higher lifetime peak BMI is associated with more frequent experiences of weight stigma and highlights peak BMI and MBS completers as predictors of higher experienced WS scores. These findings underscored the importance of incorporating comprehensive weight histories into clinical care to identify at‐risk individuals and mitigate the adverse downstream effects of experienced WS. Longitudinal studies are needed to examine how experienced WS evolves over time and whether weight loss, and its modality, mitigates or exacerbates stigma. Additionally, further research is needed to explore the racial and ethnic differences in reported Experienced WS. Addressing weight stigma through patient‐centered, evidence‐based interventions in healthcare settings may be critical for improving health outcomes and engagement for people living with obesity.

## Author Contributions

J.G., G.V., J.S., S.M. and J.A. conceived the study. J.G., G.V. and J.A. carried out the study. M.M. and J.S. acquired data. A.C. and D.E. analyzed data. All authors were involved in writing the paper and had final approval of the submitted version.

## Funding

Dr. Messiah receives funding from the National Institute of Diabetes and Digestive and Kidney Diseases (R01DK143078). Dr. Ernest is supported by the Cancer Prevention and Research Institute of Texas CIPD Training Grant (#RP210041) and University of Texas Southwestern Medical Center.

## Conflicts of Interest

Jaime Almandoz reports that he received consulting fees from AbbVie, Amgen, Boehringer Ingelheim, Eli Lilly and Company, Haleon, Kailera, Metsera, Novo Nordisk, Pfizer, Rhythm, Rivus and Wave Life Sciences.

## Data Availability

The data that support the findings of this study are available from the corresponding author upon reasonable request.
